# Efficacy of autologous stem cell-based therapy for osteonecrosis of the femoral head in sickle cell disease: a five-year follow-up study

**DOI:** 10.1186/s13287-015-0105-2

**Published:** 2015-05-29

**Authors:** Gildasio Cerqueira Daltro, Vitor Fortuna, Eliane Silva de Souza, Marcela Miranda Salles, Ana Claudia Carreira, Roberto Meyer, Songeli Menezes Freire, Radovan Borojevic

**Affiliations:** Prof. Edgar Santos Hospital Complex, Salvador, BA 40110-902 Brazil; Health Science Institute, Federal University of Bahia, Reitor Miguel Calmon Avenue, Salvador, BA 40110-100 Brazil; Cell and Molecular Therapy Center NUCEL-NETCEM, School of Medicine, Internal Medicine Department, and Chemistry Institute, Biochemistry Department, University of São Paulo, São Paulo, SP 05508-900 Brazil; Petrópolis School of Medicine/Arthur de Sá Earp Faculties, Petrópolis, RJ 25680-120 Brazil; National Institute of Metrology, Quality and Technology (Inmetro), Xerém, Rio de Janeiro, RJ 25250-020 Brazil

## Abstract

**Introduction:**

Stem cell therapy with bone marrow-derived mononuclear cells (BMMCs) is an option for improving joint function in osteonecrosis of the femoral head (ONFH). Bone marrow-derived mesenchymal stromal cell (MSC) numbers and their osteogenic differentiation are decreased in patients with ONFH. However, whether this decrease also extends to the early stages of ONFH in sickle cell disease (SCD) is still unclear.

**Methods:**

We conducted a phase I/II, non-controlled study to determine efficacy and safety of BMMC implantation using a minimally invasive technique in SCD patients with ONFH. Eighty-nine patients were recruited and followed up for 60 months after surgery. Clinical and radiographic findings were assessed, and data were completed by in vitro analysis.

**Results:**

At the final follow-up (60 months) there was a significant improvement in clinical joint symptoms and pain relief as measured by the Harris Hip Score (*P* = 0.0005). In addition, after the BMMC implantation procedure, radiographic assessment showed disease stabilization and only 3.7 % of the treated patients did not achieve a satisfactory clinical result. The amount of fibroblast colony-forming units was 28.2 ± 13.9 per 1 million BMMCs after concentration. Flow cytometry analysis showed a significantly higher number of hematopoietic stem/endothelial progenitor cell markers in concentrated BMMCs when compared with bone marrow aspirate, indicating an enrichment of these cell types. Isolated MSCs from SCD patients with pre-collapse ONFH maintained the replicative capacity without significant loss of their specific biomolecular characteristics, multi-differentiation potential, and osteogenic differentiation activities. Cytokines and growth factors (interleukin-8, transforming growth factor-beta, stromal cell-derived factor-1alpha and vascular endothelial growth factor) that mediate endogenous bone regeneration were also produced by expanded MSCs from SCD patients.

**Conclusion:**

The autologous BMMC implantation with a minimally invasive technique resulted in significant pain relief and halted the progression of early stages of ONFH in SCD patients. MSCs from SCD patients display biological properties that may add to the efficiency of surgical treatment in ONFH. In summary, our results indicate that infusion of BMMCs enriched with stem/progenitor cells is a safe and effective treatment for the early stages of ONFH in SCD patients.

**Trial registration:**

ClinicalTrials.gov NCT02448121; registered 15 May 2015.

**Electronic supplementary material:**

The online version of this article (doi:10.1186/s13287-015-0105-2) contains supplementary material, which is available to authorized users.

## Introduction

Sickle cell disease (SCD) is the most common inherited blood disease, with a worldwide distribution. In Brazil, the prevalence of hemoglobin S (HbS) carriers varies from 6 % to 15.7 % among different population groups [1]. The highest frequency of abnormal hemoglobin and the rate of race admixture, mainly of African descendent, means the presence of hemoglobinopathies is considered a public health problem in northeast Brazil [2, 3].

Osteonecrosis of the femoral head (ONFH) is a debilitating and severe complication of SCD and its treatment is still a big challenge. Depending upon the particular genotype and severity of the SCD, the prevalence of ONFH ranges from 3 to 50 % among SCD patients [4, 5].

Osteonecrosis can be viewed as a vascular and bone disease with altered bone remodeling. The combination of vascular and bone pathologies contributes to the development of osteonecrosis, which leads to inadequate bone repair that advances to subchondral fracture [6, 7]. Patients with SCD experience both large and small vessel occlusions leading to end-organ damage and complications such as ONFH. These vascular occlusion events result from various processes including hypoxia-induced erythrocyte sickling, along with extravascular compression of the intra-osseous blood supply resulting in an imbalance between osteoblast formation and necrosis, which culminates in femoral head infarction [8]. If left untreated, ONFH has a high likelihood of progression to secondary arthrosis in up to 86 % of cases [7, 9]. Once collapse occurs, total arthroplasty is a possible treatment, but its high rates of infection, medical and surgical complications, lead to overall failure rates ranging from 5 % to 63 % in SCD patients [4, 10]. Since ONFH most frequently occurs in young patients, a treatment preserving the femoral head instead of replacing it is preferable whenever possible.

Treatment of early-stage ONFH with autologous infusion of concentrated bone marrow-derived mononuclear cells (BMMC) into the necrotic lesion has been shown to be effective and safe [11, 12]. Promising results were achieved in a majority of patients when the cell therapy was applied at early stages before the collapse, to preserve the structural integrity of the subchondral plate [11, 13]. BMMCs contain bone marrow-derived mesenchymal stromal cells (MSCs) with osteogenic and chondrogenic capacity, as well as endothelial progenitor cells (EPCs) which are able to promote formation of new blood vessels and improve tissue regeneration. Mao and coworkers demonstrated that targeted delivery of autologous BMMCs enriched with MSCs could relieve symptoms, improve hip function and delay the progression of ONFH [14]. Similarly, in an experimental animal model of ONFH, Yan et al. [13] demonstrated that transplanted MSCs could differentiate into osteoblasts in the osteonecrotic region of ONFH, and secrete angiogenic factors resulting in increased angiogenesis, which in turn could contribute to the bone repair. Therefore, both the increased supply of vasculogenic and bone-forming progenitor cells [15], their homing abilities to injury sites [16, 17] and their paracrine release of a variety of growth factors [18] make the application of BMMCs with enriched stem/progenitor cell content an ideal cell-based approach to prevent fracture and collapse by restoring the architecture of the femoral head in early-stage osteonecrosis.

Among the multiple attributes of stem/progenitor cells in BMMCs, the osteogenic activity and replicative capacity of MSC are thought to play an important role in a successful therapeutic outcome in osteonecrosis. However, several reports recently suggested that MSC numbers and osteogenic differentiation abilities were impaired in patients with corticosteroid-induced and alcohol-induced ONFH [19–21]. In addition, several reports have indicated that BMMC isolation-yield and MSC replicative capacities are reduced in non-traumatic ONFH [22, 23]. A comparative analysis of the osteogenic activity and growth potential of MSCs in patients with ONFH has shown differences according to the risk factors [21], but whether this impairment also extends to stem/progenitor cells from the early stages of ONFH related to SCD is still unclear.

The 1-year results of the observational pilot study on the efficacy and safety of BMMC implantation suggested that cell-based therapy could improve joint symptoms and delay disease progression in our population [24]. However, these results needed to be confirmed by the 5-year follow-up to emphasize their clinical relevance. We will present here the 5-year results to investigate the efficacy and safety of autologous BMMC implantation combined with a minimally invasive decompression technique as a treatment for early stage ONFH in SCD patients. Our clinical and radiographical findings were complemented with promising in-vitro data. Because there is a paucity of recent MSC reports in patients who have sickle cell hemoglobinopathy, we undertook this study to evaluate whether the biological properties of human MSCs support the therapeutic potential of enriched BMMCs in SCD.

## Material and methods

### Study design

This single center prospective study was conducted in the university-affiliated Professor Edgard Santos Hospital (HUPES, UFBA) in Salvador (BA), Brazil. The Committee of Ethics in Research of the Climério de Oliveira Maternity and the National Committee of Ethics in Research (CONEP) approved the protocol of the present study under the authorization of the Ministry of Public Health of Brazil (registration number 11738, SIPAR/MS: 25000.039812/2005–99). Written informed consent was obtained from every patient before enrollment in the study.

### Participant selection

From 2006 to 2013, 89 patients were submitted to implants of BMMCs according to the technique described by Hernigou and Beaujean [11]. Inclusion criteria for SCD patients with early stages (pre-collapse) of ONFH were those of conventional core decompression surgery (that is, radiological diagnosis of ONFH according to Ficat stage 0, I, IIA and IIB or Steinberg classification A, B or C; see Additional file 1) [25, 26], and scoring at least 20 points on the pain and daily life activities questionnaire. The exclusion criteria were patients under 18 years of age, those with Ficat stage III and Steinberg’s C (or more) ONFH, current and previous bone infection at the limb affected by necrosis, acute recurrent painful crises, immunosuppressive drug therapy, a history of previous surgery on the same injured limb, prior systemic corticosteroid treatment, pregnancy, presence of neoplastic disease or any other clinical concurrent condition other than SCD that predisposed them to the development of osteonecrosis of the femoral head (e.g., a history of chronic systemic steroid use or alcoholism) or contraindicating the procedure.

The hematology analyses of patients were carried out in an automatic Coulter T 890 device (Beckman Coulter, Inc., Brea, CA, USA). SCD diagnosis and the hemoglobin analyses were confirmed by cellulose acetate electrophoresis at alkaline pH [27]. A total of 41 men and 48 women were included in this study. The mean age of patients at the time of treatment was 33 years (18 to 55 years). Among them, 61.8 % were HbSC, sickle-hemoglobin C disease patients and 38.2 % were HbSS, sickle cell anemia patients.

### Clinical and radiographic evaluation

Two experienced orthopedic surgeons assessed the clinical outcomes of all patients. Patients were evaluated preoperatively, and 3, 6, 12, 24, 36, 48 and 60 months postoperatively, or whenever necessary. Patient’s pain (44 points), joint function (47 points), joint range of motion (5 points), and deformities (4 points) were recorded according to Harris Hip Score (HHS) system as 0 to 100 points. HHS was obtained preoperatively and at each follow-up visit. Outcomes were graded as excellent when the HHS was greater than 90 points, good when it was between 80 and 89 points, fair when it was between 70 and 79 points, and poor when it was less than 70 points, according to previous reports [28, 29]. Each hip of patients suffering from bilateral hip involvement was examined separately. Complications of the treatment were registered at each follow-up. Patients that developed a superficial skin or wound infection were treated successfully non-operatively with antibiotics. Efficacy of the procedure was evaluated via: (1) improvement in pain and range of motion - HHS was compared to the score before surgery; (2) radiological progression; and (3) the need for further surgery or hip replacement.

ONFH diagnosis was determined by X-ray image examination at the antero-posterior and lateral planes. When SCD patients had moderate to severe pain localized in the hip or inguinal area, and physical examination showed limitation of movement of the affected hip while X-ray showed no changes, the diagnosis of ONFH was made by magnetic resonance imaging (MRI) at the onset of treatment. Radiological progression was decided according to the development of the Ficat stage and Steinberg classification aiming to determine the following characteristics: presence of femoral head collapse; presence of a sclerosis range on femoral head; crescent sign; double signal at T2 on MRI; cyst or sclerosis on femoral head; hip pain at motion; low-intensity focus on T1.

### Bone marrow cell collection and concentration

A total of 120 mL bone marrow aspirate (BMA) was obtained by puncture and aspiration of the posterior iliac crest of patients under general anesthesia. The marrow was aspirated in small fractions at a time (10 mL heparinized plastic syringe) to reduce the degree of dilution by peripheral blood and to maximize the number of progenitors in the graft site [30]. BMMCs were concentrated in the SEPAX cell separator (Biosafe, Eysins, Switzerland) in accordance with the manufacturer’s recommendations. After centrifugation, a final volume (~40 mL) of BMMCs in suspension with 5 % human serum albumin was harvested and placed in syringes for re-injection during the same operation. A small fraction of the BMA and BMMCs were separated for flow cytometry, cell viability and microbiological assays.

### BMMC grafting

The concentrated BMMC was injected through a percutaneous approach using a 3-mm diameter trocar (trocar of Mazabraud, Collin, France) in the center of the osteonecrotic area. The procedure was previously planned using nuclear magnetic resonance images, and the needle position at the femoral head area was monitored by fluoroscopy. Each patient received the BMMC infusion through a single injection puncture. A small local blood tampon avoided leakage of the infused BMMC fraction.

### Clonal BMMC assays

Colony-forming unit fibroblast (CFU-F) assays were performed to determine the number of mesenchymal progenitors in BMMC samples, as described previously [31]. Briefly, single cell suspensions of BMMCs were cultivated at 1.0 × 10^6^ cells/25 cm^2^ flasks with Dulbecco’s modified Eagle’s medium (DMEM) low glucose (Sigma, St. Louis, MO, USA), supplemented with 20 % fetal bovine serum (Cultilab, Campinas, Brazil), 100 U/mL penicillin and 100 μg/mL streptomycin (Invitrogen, Grand Island, NY, USA) and incubated in 5 % CO_2_ at 37 °C. Medium was renewed every third day. After 10 days, the non-adherent cells were removed by washing with phosphate-buffered saline and the remaining adherent cells were fixed in 4 % buffered formalin for 10 minutes. To detect CFU-F, the cells were stained with Crystal Violet solution. The cultures were scored for clusters (20–50 cells/aggregate) and colonies (>50 cells) at 10× magnification microscopy. To determine the colony-forming unit osteoblast (CFU-O) potential of BMMCs, cells were cultivated in basal media containing 10 nM dexamethasone (Sigma), 5 mM sodium β-glycerophosphate (Sigma), and 50 mg/mL ascorbic acid-2-phosphate (Sigma) and plated as described [32]. After 21–24 days in culture, CFU-O were fixed in 4 % buffered formalin for 10 minutes and stained with Alizarin S for mineralization (see below).

### Immunophenotyping

BMMCs and BMA were analyzed for the expression of cell-surface antigens with direct three-color analysis using fluorescein isothiocyanate (FITC)-conjugated, phycoerythrin (PE)-conjugated and peridinin chlorophyll protein complex (PerCP)-conjugated monoclonal antibodies as previously reported [33, 34]. The following antibodies were used for analysis: immunoglobulin G1 control PE (eBioscience, San Diego, CA, USA), immunoglobulin G1 control PerCP (Becton Dickinson, San Jose, CA, USA), immunoglobulin G1 control FITC (BD Bioscience, Franklin Lakes, NJ, USA), anti-CD34-FITC (BD Bioscience), anti-CD34-PerCP (Becton Dickinson), anti-KDR-PE (R&D Systems, Minneapolis, MN, USA), anti CD45-PerCP (BD Bioscience), and anti-CD133–PE (Miltenyi Biotec, Bergisch Gladbach, Germany). The quantification of total mononuclear cells in BMMCs and BMA was determined by Turk’s solution. For FACS analysis, 5 × 10^5^ events were acquired and scored with a FACSCalibur analyzer (Becton Dickinson). Data were processed using the Macintosh CELLQuest software program (Becton Dickinson).

### BMMC viability and quality control

The viability of infused cells, as determined by Trypan blue exclusion, was above 95 %. The study of the presence of potential microbiologic contaminants, monitored by blood culture and traditional microbiologic tests, showed negative results.

### Isolation and culture of primary human MSCs

BMMCs were plated at a density of 1.6 × 10^5^ mononucleated cells/cm^2^ in DMEM low-glucose as described above. After 4–5 days of culture, the non-adherent cells were discarded and the medium was refreshed twice a week. On reaching 90 % confluence, the cells were sub-cultured using 0.05 % trypsin–0.02 % EDTA at a 1:3 ratio. MSCs were used up to passage 6 for all studies.

### Immunophenotypic characterization of MSCs

The expanded MSC population at third passage in cell culture were characterized by morphology (spindle-shaped cells) and by immunophenotypic analysis for the expression of the following membrane markers: mouse anti-human CD14-PE (clone 61D3), CD29-FITC (clone TS2/16), CD90-FITC (clone eBIO5E10) and CD105-PE (clone SN6) (from eBioscience), CD31-FITC (clone WM59), CD34-FITC (clone 8G12), CD45-PerCP-Cy5.5 (clone MOPC-21) and CD146-PE (clone P1H12) (from BD Biosciences, San Jose, CA, USA). Detection of osteocalcin in MSC culture by flow cytometry was performed with mouse anti-human PE-conjugated monoclonal antibody (R&D system) or isotype control antibody according to the manufacturer’s protocol.

### Osteogenic differentiation of MSCs and matrix quantification

For osteogenic differentiation, 5.0 × 10^4^ MSCs were plated in a 24-well plate in triplicate. Osteogenic differentiation was induced by culturing confluent MSCs in DMEM basic medium (4 mmol/L L-glutamine/penicillin/streptomycin, 10 % fetal bovine serum) supplemented with 100 nmol/L dexamethasone (Sigma), 5 mM sodium β-glycerophosphate (Sigma), and 50 μg/mL ascorbic acid 2-phosphate (Sigma) for 21 days, with media change every 3–4 days. After differentiation, cells were fixed for 10 minutes with 70 % ice-cold ethanol and subsequently stained with Alizarin Red (Sigma; 1 % Alizarin Red in distilled water, pH 4.2) to indicate calcium deposition and mineralized nodules within the extracellular matrix [21]. Morphological evaluation of cells was performed using a DM IL LED inverted microscope (Leica, Wetzlar, Germany) coupled to a digital camera for imaging capture. Quantitative analysis of the amount of Alizarin staining was performed with Fiji (NIH, Bethesda, Maryland, USA). Five fields were selected and the newly formed mineralized tissue area in each field was calculated and shown as a percentage of total tissue area.

### Adipogenic differentiation of MSCs

MSCs were seeded into 24-well tissue-culture plates at a density of 5.0 × 10^4^ cells/well. Adipogenic differentiation was induced in confluent MSC incubated with DMEM basic medium supplemented with 10 μg/mL insulin, 500 μmol/L 3-isobutyl-1-methylxanthine, 100 μmol/L indomethacin and 1 μmol/L dexamethasone. After 3 weeks of adipogenic stimulation, cells were fixed with 4 % paraformaldehyde for 30 minutes, washed with sterile water, and incubated with 0.5 % oil red O in isopropanol for 20 minutes at room temperature. After staining, cells were washed with 60 % isopropanol and rinsed with sterile water, before observing under the microscope for imaging [21].

### Chondrogenic differentiation

Chondrogenic differentiation was carried out using micromass cell cultures under chondrocyte differentiation medium (Invitrogen) for 30 days. One million MSCs were pelleted at 300 g and chondrocyte differentiation medium was added without disturbing the pellet. Media was changed every 48 hours. After differentiation, cells were fixed with 4 % paraformaldehyde and embedded in paraffin. Deparaffinized 5 μm sections were stained for proteoglycans using 3 % Alcian blue. After staining, sections were rinsed with distilled water, air dried at room temperature, immersed in xylene, and mounted for microscopy. Images were taken with a Nikon Ti Eclipse microscope (Tokyo, Japan).

### Quantitative RT-PCR

To determine the relative expression levels of the osteogenic markers, 3.0 × 10^5^ MSCs were seeded onto 25-cm^2^ flasks and maintained in control conditions or osteogenic differentiation medium. After 7 days, cells were washed with ice-cold phosphate-buffered saline, and total mRNA was isolated using silica columns from the RNeasy® mini-kit (Qiagen, Hilden, Germany), according to the manufacturer’s protocol. The mRNA concentration was determined by absorbance at 260 nm and the purity of the preparations was evaluated by the A260nm/A280nm ratio using a Nanodrop ND-1000 spectophotometer (NanoDrop Technologies, Rockland, DE, USA). cDNA was synthesized from 1 μg total RNA using Superscript II reverse transcriptase (Invitrogen), followed by RNA digestion with RNase H (Invitrogen). cDNA purity was confirmed by PCR for glyceraldehyde-3-phosphate dehydrogenase (GAPDH).

Quantitative RT-PCR was carried out using the ABI Prism 7000 sequence detection system (Applied Biosystems, (Foster City, CA, USA). All samples were run in triplicate in 96-well plates. The quantitative RT-PCR reaction was carried out using 6 μl SYBR® Green Dye (Applied Biosystems), 3 μl of 30-times diluted cDNA and 3 μl of a mix containing both the forward and the reverse primers (see Additional file 2), and incubated under the following conditions: 2 minutes at 50 °C, 10 minutes at 95 °C, followed by 40 cycles of 15 s at 95 °C and 60 °C for 1 minute. GAPDH, HPRT, and RN18S1 were used as reference genes. Relative expression levels of each gene were analyzed using the 7300 System SDS Software (Applied Biosystems) with the 2^-ΔΔCt^ method [35]. The data are shown as mean 2^-ΔΔCt^ ± standard error. The experiment was repeated using MSCs from three different donors.

### Cytokine assays

MSCs (3.0 × 10^4^/cm^2^) were seeded in 24-well culture plates. After 48 hours the supernatants were collected and the levels of transforming growth factor (TGF)-β (Invitrogen), interleukin (IL)-8 (Invitrogen), vascular endothelial growth factor (VEGF; R&D System) and stromal cell-derived factor (SDF)-1 alpha (R&D System Inc) were measured by enzyme-linked immunosorbent assay kits according to the manufacturer’s instructions.

### Statistical analysis

The paired *t*-test for variance analysis was employed to determine the significance level of the differences found between pre- and postoperative HHS values. Nonparametric tests were used as the statistical method for in vitro experimental data because most of the variables were small numbers. Correlation coefficients between clinical assessment results and cell factors were determined by means of the Spearman’s correlation test. The results are expressed as the mean values ± standard deviation. All statistical analyses were performed using GraphPad Prism software (Version 6.0; GraphPad Software, La Jolla, CA, USA). A value of *P* < 0.05 was considered significant.

## Results

### Improved clinical outcome of femoral heads treated with BMMCs at early stages of osteonecrosis in sickle cell disease

In the present study, we followed-up 89 SCD patients with ONFH. Our study was limited to autografts of BMMCs for patients with pre-collapse osteonecrosis of the femoral head (Ficat stage 0, I or II). The mean length of follow-up was 37.3 months (range 12 to 60 months). No complications such as fracture, dislocation, nerve or muscle lesions, clinically significant thromboembolism or hematoma were observed during or after the treatment. No patient has required further surgery.

**Fig. 1:**
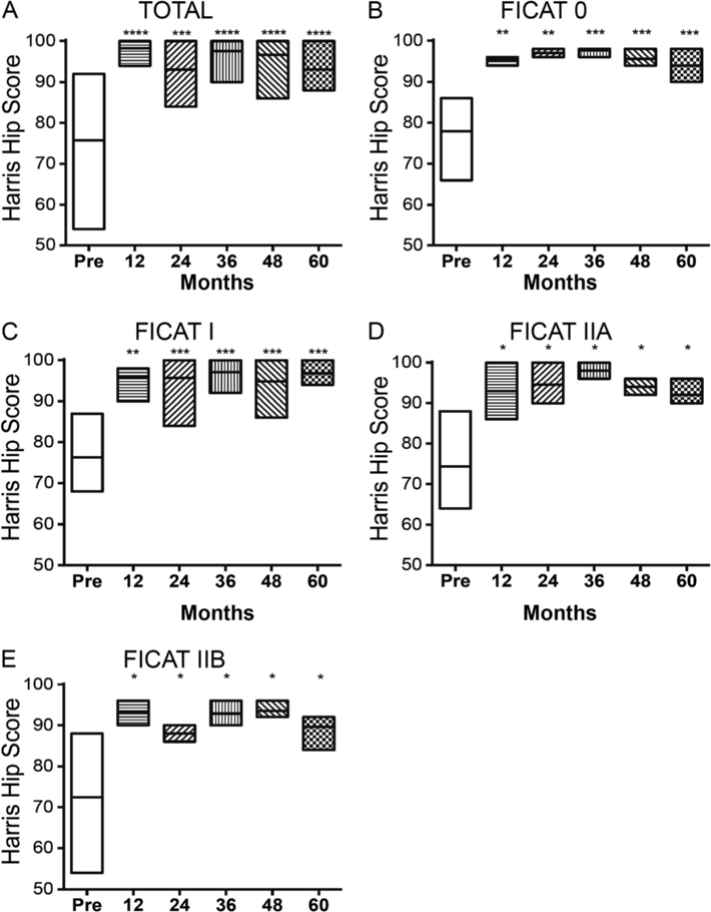
BMMC treatment significantly improved the outcome of femoral heads at early-stage ONFH in SCD. **a**–**e** Changes in HHS for femoral heads grouped according to Ficat stage over the entire postoperative period. Floating bars represent maximum and minimum range of variation, with lines at mean HHS. *P < 0.05, **P < 0.01, ***P < 0.001, ****P < 0.0001, versus preoperative HHS values

The preoperative HHS for the cohort was 75.7 points, and the mean score at the most recent follow-up was 93.1 points, indicating a significant difference before and after operation (*P* = 0.0005) (Fig. 1a). Our procedure achieved a significant increase in the average HHS for the entire group of patients with pre-collapse stages of the disease, as compared to HHS before treatment (Fig. 1b-e). Over the 60-month follow-up period, the mean HHS improved from preoperative 77.9 points (range 66–86 points) to postoperative 94.0 points (range 90–98 points) for femoral heads with Ficat stage 0 (*P* = 0.0009, 95 % confidence interval; Fig. 1b). For those with Ficat stage I, mean HSS improved from preoperative 76.3 points (range 68–87 points) to postoperative 96.8 points (range 94–100 points) (*P* = 0.0003, 95 % confidence interval; Fig. 1c). For femoral heads with Ficat stage IIA, mean HSS improved from preoperative 71.8 points (range 64–88 points) to postoperative 92 points (range 90–96 points) (*P* = 0.028, 95 % confidence interval; Fig. 1d). For femoral heads with Ficat stage IIB, mean HSS improved from preoperative 70.9 points (range 54–88 points) to postoperative 89.6 points (range 84–92 points) (*P* = 0.013, 95 % confidence interval; Fig. 1e). The mean HHS before and after implantation of BMMCs is summarized in Table 1. These significant differences indicated the beneficial effect of BMMC infusion on the recovery of hip function, reduced pain, and higher quality of daily activities of the patients.

**Table 1:** Stratified Harris Hip Score before and after implantation of bone marrow-derived mononuclear cells

	Mean Harris Hip Score (mean and range)		
Ficat and Arlet stage (n = patients)	Preoperative	Final follow-up	*P* value
Stage 0 (n = 20)	77.9 (66–86)	94.0 (90–98)	0.0009
Stage I (n = 31)	76.3 (68–87)	96.8 (94–100)	0.0003
Stage IIA (n = 16)	71.8 (64–88)	92.0 (90–96)	0.028
Stage IIB (n = 22)	70.9 (54–88)	89.6 (84–92)	0.013
Total (n = 89)	75.7 (54–92)	93.1 (88–100)	0.0005

Forty-five days after the procedure, 60 patients reported no pain and 26 patients reported moderate pain, which disappeared by 90 to 100 days. Considering clinical improvement in pain, only 3.7 % (3 out of 89) of the treated patients did not reach a satisfactory clinical result. Two of 16 patients with a femoral head with Ficat stage IIA reported no decrease in pain at 10 to 12 months after surgery. One of 16 patients with a femoral head at Ficat stage IIA reported progressive and severe hip pain at 18 months. However, at X-ray examination the disease stage of these femoral heads remained unchanged. During the follow-up period, the patients showed no radiographic progression of the disease.

### Radiographic outcome

Preoperative and postoperative X-ray evaluations of patients indicated that the radiographic aspects were less significant than the clinical evaluation results. Antero-posterior X-ray projections of hips whose clinical progress was favorable did not show any evolution to subchondral fracture in the weight-bearing area (curvilinear subchondral radiolucent line) or disease progression, with no patent enlargement of the lesion size. Postoperative MRI scans demonstrated changes in lesion size and signals due to reduction of bone-marrow edema surrounding the necrotic lesion. Most lesions that appeared stable on MRI were clinically also stable or improved.

**Fig. 2:**
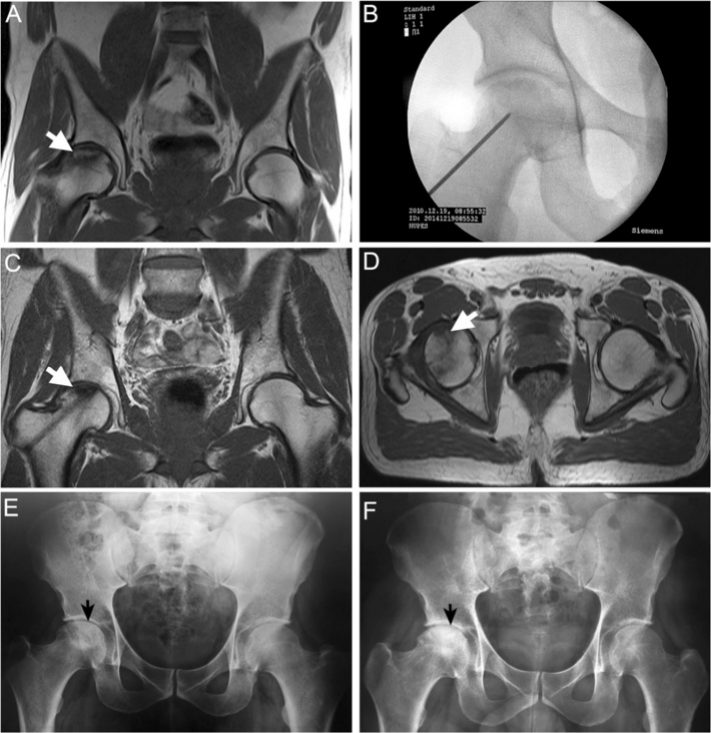
Radiographic sequence from a representative case of a symptomatic 44-year-old man with ONFH successfully treated with BMMC implantation. **a** Coronal T1-weighted MRI scans of the right hip before surgery show osteonecrotic lesion in Ficat IIB stage. The osteonecrotic lesion is surrounded by sclerotic low-signal margin (*arrow*) with subjacent marrow edema. **b** Anteroposterior X-ray radiograph showing minimally invasive decompression. The trephine was introduced under fluoroscopy through the greater trochanter into the femoral head. The BMMCs were injected through the trephine into the necrotic zone. **c, d** Twelve-month postoperative coronal (c) and axial (d) MRI (T1-weighted) scans after autologous BMMC implantation showing the channel, decreased bone edema and changes of signal in the superior part of the femur. Axial MR image (d) shows well-delimited marrow region of increased signal intensity (*arrow*) within the necrotic area and hypointense line around them due to reparative new bone. **e, f** Preoperative (e) and 60-month postoperative anteroposterior (f) radiographs of the same hip showing the femoral head has maintained the sphericity (*arrow*)

Radiographic images from a representative successful case of a SCD patient with stage IIB osteonecrosis of the femoral head, successfully treated with BMMC implantation at 60-month follow-up period, are shown in Fig. 2.

### Grafted BMMCs enriched in connective and osteogenic progenitor cells

**Fig. 3:**
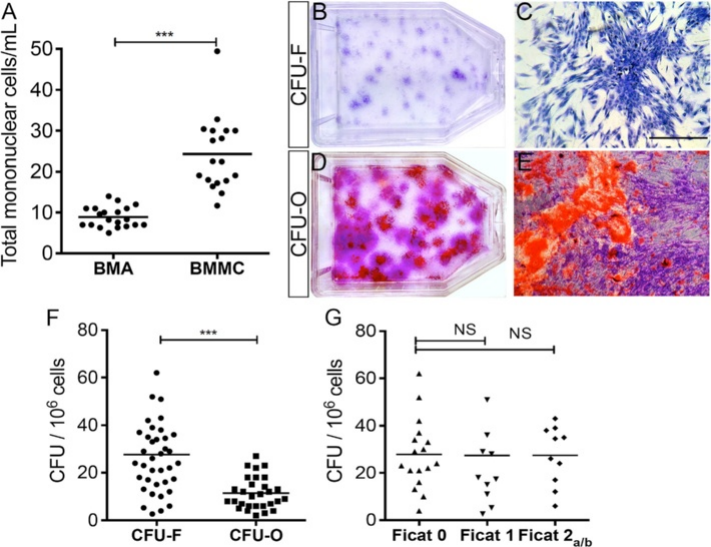
Increased number of conjunctive and osteogenic progenitor cells in concentrated BMMC. **a** Scatter plots showing the distribution of total mononuclear cell number in 1 × 10^6^ cells/mL after bone marrow aspiration (*BMA*) and after concentration of bone marrow-derived mononuclear cells (*BMMC*) on Ficoll density gradient centrifugation. **b, c** Flask and morphologic characteristics of colony-forming unit-fibroblasts (*CFU-F*) from BMMC. **d, e** Flask with mineralized matrix stained with red Alizarin-S and Crystal Violet of colony-forming unit-osteoblasts (*CFU-O*) from BMMC. **f** Frequency of CFU-F/10^6^ cells and CFU-O/10^6^ cells in concentrated BMMC. **g** Frequency of CFU-F/10^6^ cells isolated in BMMC from individuals with osteonecrosis at a distant site was independent of the disease stage. The high standard deviation indicates the high variability of CFU-F and CFU-O within the SCD bone marrow. Data are represented as individuals values. Horizontal bars indicate mean value. Scale bar: 50 μm. ***P < 0.001. *NS*, not significant

An average of 120 ± 10 mL of bone marrow harvest from the iliac crest was concentrated into 40 ± 8 mL within 90 minutes after density centrifugation with Ficoll. The number of mononuclear cells increased from 8.9 ± 2.6 × 10^6^ cells/mL in the initial BMA to 24.3 ± 9.1 × 10^6^ cells/mL after concentration (Fig. 3a). After 10 days in culture, an average of 28.2 ± 13.9 CFU-F colonies was observed in BMMC (1.0 × 10^6^ cells/25 cm^2^) cultures (Fig. 3b, c, f) compared to 8.4 ± 5.3 colonies (1.0 × 10^6^ cells/25 cm^2^) in the BMA group (see Additional file 3). A final volume of 40 mL concentrated BMMC suspension was injected into the femoral head; hence, we have administered an average of 9.7 ± 3.6 × 10^8^ BMMCs to each femoral head with ONFH which contained about 2.7 ± 1.4 × 10^4^/cells of CFU-F.

After 21 days under osteogenic medium cultivation, bone nodules were detected with Alizarin Red stain, indicating the presence of osteoprogenitor cells (CFU-O) in BMMC (Fig. 3d–f). The frequency of CFU-O represented approximately 40 % of CFU-F (Fig. 3f) demonstrating that our technique isolates and concentrates a heterogeneous mixture of mononuclear cell populations especially rich in connective tissue and osteogenic stem cells. Of importance for their clinical utility, the total amount of CFU-F and morphological findings were not statistically different among the group of SCD patients with pre-collapse ONFH (Fig. 3g). Moreover, these results demonstrate that the grafted BMMC contained viable progenitor cells even when the injection was performed 1 hour after the initial aspiration, during our surgical technique.

### Endothelial progenitor/hematopoietic stem cell phenotype frequencies are increased in grafted BMMCs

In order to further characterize the heterogeneous group of the BMMC population, the phenotype of BMA and BMMC was analyzed for the surface markers: CD34, CD133, KDR (VEGFR2), CD45 and their appropriate isotype-matched IgGs. EPCs were predefined as cells expressing a combination of surface markers CD34^+^CD133^+^KDR^+^, to indicate that they belong to the bone marrow early progenitors (CD34^+^), their immaturity (CD133^+^) and endothelial origin (KDR^+^). Conversely, another subset of CD45^dim^ cells indicated immaturity/hematopoietic origin, marking the phenotype CD34^+^CD45^dim^KDR^+^ as a different subpopulation of EPC.

**Fig. 4:**
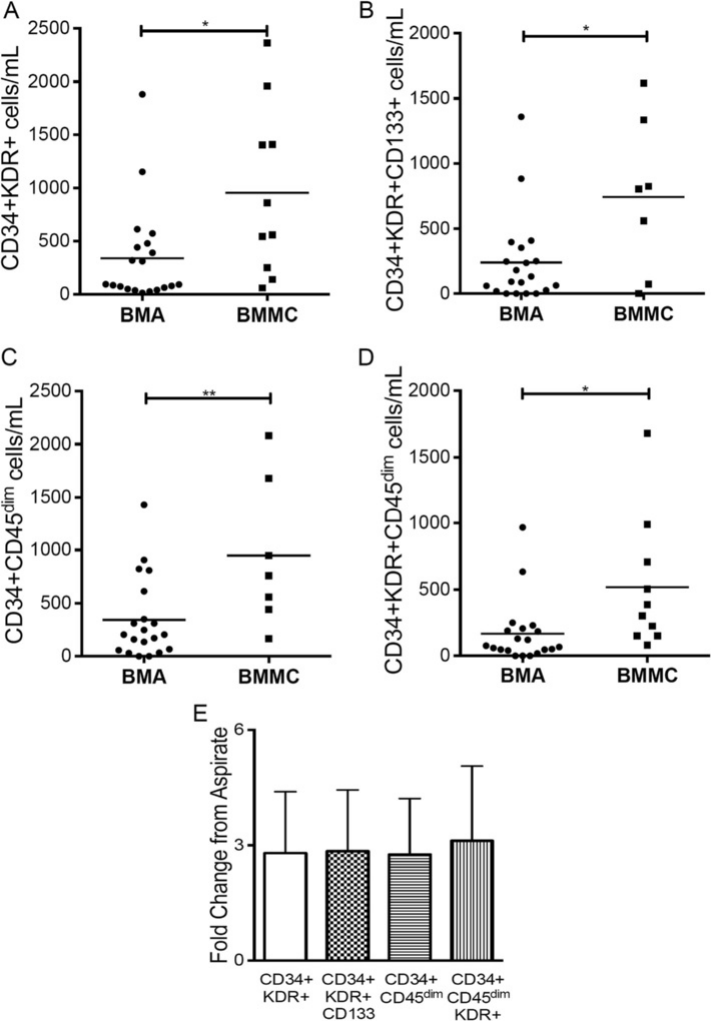
Concentrated number of endothelial and hematopoietic progenitor cell phenotypes in BMMC. **a–d** The frequency of cell subpopulations was obtained after labeling of total bone marrow-derived mononuclear cells (*BMMC*) (1 × 10^6^ cells) separately with antibodies (set 1 CD133/CD34/KDR or set 2 CD34/KDR/CD45) and analysis by flow cytometry. Phenotypes CD34^+^/KDR^+^ **(a)**, CD34^+^/KDR^+^/CD133^+^ **(b)**, CD34/CD45^dim^ **(c)** and CD34^+^/KDR^+^/CD45^dim^ (d) were concentrated in the implanted BMMC of SCD patients. **e** Concentration factor calculated by the quotient of the specified phenotype number in BMMC/BMA. The high standard deviation indicates the high variability of cell phenotypes within the SCD bone marrow. Data are represented as individuals values. Horizontal bars indicate mean value. *P < 0.05, **P < 0.01, ***P < 0.005, ****P < 0.001. *BMA* bone marrow aspirate

There were significantly lower quantities of CD34^+^CD133^+^KDR^+^ (*P* < 0.01) and CD34^+^CD45^dim^KDR^+^ (*P* < 0.01) in BMA as compared to in BMMCs, showing that fresh EPC were enriched in the grafted BMMCs (Fig. 4a–d). In addition to the EPC phenotype, we also found CD34^+^/CD45^dim^ (*P* < 0.007) to be significantly different between BMA and BMMCs. The average concentration of BMMCs compared to the initial BMA was 2.8 ± 1.4-fold for CD34^+^KDR^+^, CD34^+^CD133^+^KDR^+^, CD34^+^CD45^dim^ and 3.2 ± 1.5-fold for CD34 + CD45^dim^KDR^+^ (Fig. 4e).

Overall, EPC identifying markers (CD34, KDR, CD133 and CD45^dim^) were significantly enriched after separation on BMMCs as compared to the fresh BMA samples. The numbers of cell phenotypes for various marker combinations are shown in Table 2.

**Table 2:** Endothelial progenitor cell phenotype and different progenitors are enriched in bone marrow-derived mononuclear cells (number of cells/mL ± SD)

Cell phenotype	BMA (n = 20)	BMMC (n = 10)	*P* value
CD34^+^CD133^+^	587.9 ± 239.4	756.9 ± 269.0	0.667
CD34^+^KDR^+^	341.0 ± 103.5	954,9 ± 251.7	0.012
CD34^+^CD133^+^KDR^+^	239.6 ± 75.75	744.9 ± 226.7	0.010
CD34^+^CD45^dim^	342.9 ± 85.17	947.4 ± 261.7	0.007
CD34^+^CD45^dim^KDR^+^	166.2 ± 53.04	517.8 ± 157.0	0.013

*BMA* bone marrow aspirate; *BMMC* bone marrow-derived mononuclear cells

### Multipotent mesenchymal stem cell phenotype is present in BMMCs from sickle cell disease patients

**Fig. 5:**
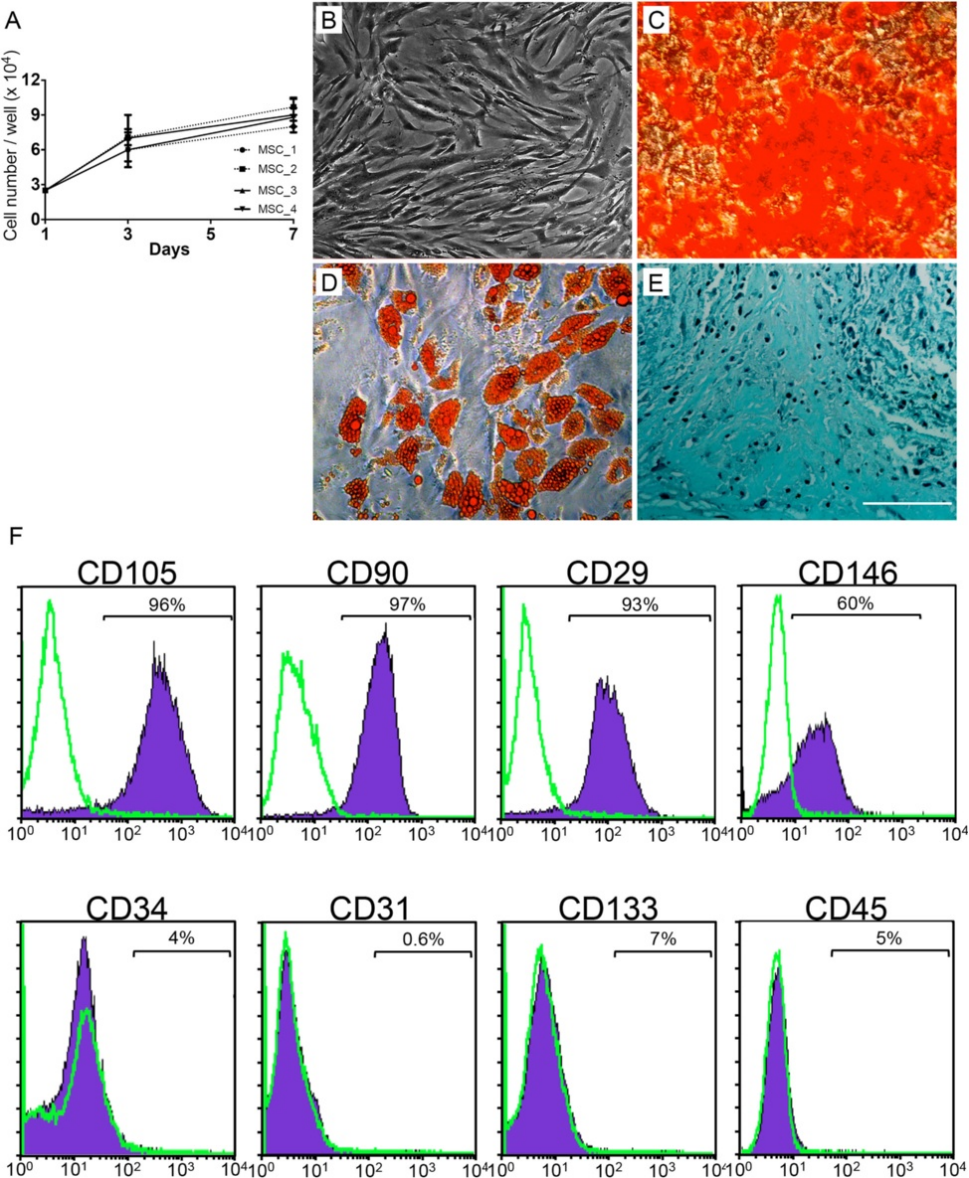
Expansion, characterization and pluripotency properties of human MSCs from SCD patients. **a** Ex vivo expanded mesenchymal stromal cells (*MSC*) from SCD patients with pre-collapse ONFH at a distant site in control culture conditions. Total viable cell counts of MSCs from donors with progressive disease stage are plotted for MSCs expanded in fetal bovine serum. **b** Morphologic characteristics of MSCs at passage 3. **c**–**e** Differentiation of MSCs. Results of staining assays on MSCs induced to osteogenic differentiation (c, mineralized bone nodules stained with Alizarin red), adipogenic differentiation (d, intracellular lipid droplets stained with Oil red O), and chondrogenic differentiation (e, static micromass cell culture stained with Alcian Blue). **f** Immunophenotyping profile of MSCs isolated from SCD patients. Negative peaks (*green line*) correspond to cell autofluorescence. MSCs were positively labeled to CD29, CD90, CD105, CD146 and negatively labeled to CD31, CD34, CD45 and CD133. Data are mean ± SD number of cells (n = 4). Data are representative of three independent experiments. Magnification: 400× in b–d. Scale bar: 50 μm in **e**

Previous studies have described that the differentiation potential, proliferation rate and MSC content in BMMCs were variable in patients with non-traumatic osteonecrosis [19–21], suggesting that the effectiveness of cell therapy may be related to the availability of MSCs according to the risk factor. In view of a paucity of information regarding MSCs in SCD, we decided to investigate the biological functions of MCSs in these patients with pre-collapse osteonecrosis. First, we selected age-matched and Ficat stage-matched BMMC samples to isolate MSC populations. The expansion kinetics curves and the morphological characteristics of ex vivo MSCs were not statistically different among SCD patients with pre-collapsed ONFH. Expanded MSC from SCD patients formed a homogenous monolayer of adherent spindle shaped fibroblast-like cells (Fig. 5a, b). The isolation protocol proved to be successful in all the BMMC collections, and sufficient numbers of MSCs were obtained after 4 weeks. After the third passage, we observed for each culture that isolated cells were negative for typical hematopoietic or endothelial antigens CD45, CD34, CD133 and CD31, and were positive for mesenchymal cell surface markers CD29, CD90, CD105, CD146 (Fig. 5f). Expanded MSC populations at low passages (third to sixth) showed a similar expression pattern of surface markers. In addition, there was no significant variation of surface marker expression in MSC cultures from different hemoglobin genotype or disease stage samples (see Additional file 4).

### Osteogenic potential of MSCs is not defective in sickle cell disease patients with osteonecrosis

The differentiation of MSCs from SCD patients into adipogenic, osteogenic and chondrogenic phenotypes was monitored using specific staining as previously described. All MSC cultures showed the capacity to differentiate along the three lineages tested (Fig. 5c–e). After adipogenic differentiation, lipid droplets developed inside the cell cytoplasm and were stained by Oil-Red-O, which proved adipogenic differentiation in MSC expanded samples (Fig. 5c). After the induction of chondrogenesis, the samples were positively stained for acid mucopolysaccharides by Alcian blue (Fig. 5e). After osteogenic induction, cell shape flattened and broadened (Fig. 5d). Osteogenic differentiation was associated with calcium deposit formation, and staining by Alizarin Red S was found in all MSC samples. The differentiation potential of MSCs was maintained up to 6 passages. These in vitro differentiation assays confirmed the multipotent potential of MSCs present in BMMC from SCD patients.

**Fig. 6:**
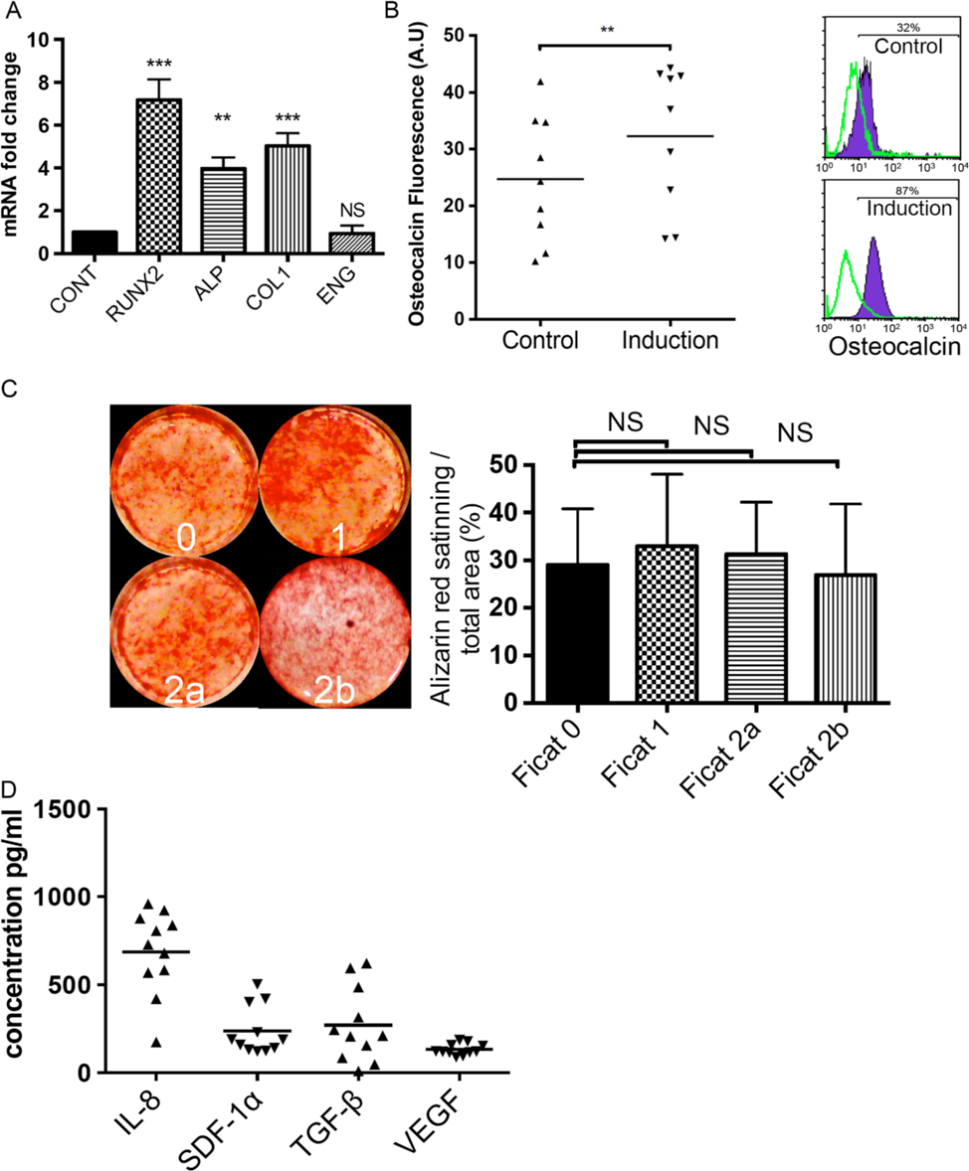
Osteogenic abilities of bone marrow-derived MSCs are not defective in patients with osteonecrosis. **a** Quantitative RT-PCR of osteoblast-related genes from control and osteogenic-induced MSCs after 10 days were normalized and compared according to housekeeping genes as control. **b** Scatter plots (*left*) showing the distribution of total fluorescence intensity from FACS analysis (*right*) of cellular osteocalcin expression in MSCs. The relative fluorescence intensity shows significant differences between control and osteogenic differentiation conditions. **c** Alizarin Red staining (*left*) indicating mineralized nodule formation of cultured MSCs after treatment with osteogenic media for 21 days. Quantitative analysis of the amount of Alizarin staining in each group is also indicated (*right*). No significant difference (*NS*) was found between MSCs from SCD patients with early-stage osteonecrosis and patients with further stages of the disease at a distant site. Alizarin Red staining was independent of the disease stage. **d** Enzyme-linked immunosorbent assay for interleukin-8 (*IL-8*), transforming growth factor-beta (*TGF-β*), stromal cell-derived factor-1alpha (*SDF-1α*) and vascular endothelial growth factor (*VEGF*). Concentration (pg/mL) of cytokines and growth factors in supernatants of MSCs of SCD patients after 2 days of culture. MSCs from SCD patients produce distinct levels of IL-8. Horizontal bars represent the mean and SD of data. The triangles represent individual values. *P < 0.05, **P < 0.01, ***P < 0.001

The immunocytochemical staining results for osteogenic induction were confirmed by quantitative PCR. Osteoblast lineage-specific markers (RUNX2, ALPL and COL1) are expressed during in vitro osteodifferentiation of MSCs and precede the late-stage mineralization process [36, 37]. After 10 days of differentiation, significantly higher expression levels of osteogenic specific genes RUNX2, ALPL and COL1 were observed in MSCs cultured under osteogenic medium, as compared with control MSC samples from at least five SCD patients (Fig. 6a). Quantitative PCR analysis showed that mRNA fold-changes of the osteogenic genes were highly similar in differentiated MSCs, with the exception of ENG mRNA expression levels, which were similar between control and osteogenic-induced samples (Fig. 6a). Expressions of genes specific for osteogenesis were not significantly different between samples from HbSS and HbSC. Osteocalcin, a marker of developing osteoblasts, was detected at higher levels in osteogenic-induced MSCs compared to control MSCs after 10 days (Fig. 6b). The upregulation of osteogenesis-specific genes had already started after 10 days of differentiation, although no calcium formation was observed at this time point. However, after 21 days, the mean percentage of the area stained with Alizarin Red S was 28.3 ± 10.5 % in MSCs cultured under osteogenic medium, as compared with control MSC samples from SCD patients with ONFH. The amount of mineralized matrix deposited during in vitro osteogenic differentiation was not statistically different among MSCs of SCD patients with pre-collapse osteonecrosis stages (Fig. 6c). These results indicated that ex vivo MSCs isolated from SCD patients maintain the ability of osteogenic differentiation after population expansion in our culture conditions.

The observation that MSC populations can be expanded from SCD patients with osteonecrosis and can readily be induced to an osteogenic phenotype does not exclude the possibility that they may also produce and secrete amounts of growth factors and cytokines that mediate endogenous bone regeneration. Therefore, we analyzed the protein levels of IL-8, TGF-β, SDF-1α and VEGF-A in the conditioned culture media of MSCs as these proteins are known to be intimately involved in the regulation of bone metabolism. We found that MSCs grown under control conditions secreted significant amounts of IL-8, TGF-β and SDF-1α, although with different levels (Fig. 6d). Cell culture medium without cells or serum was used as the negative control. Based on the observation that osteogenic differentiation of MSCs was not hampered under our culture conditions, we questioned whether MSCs secreted factors that might improve vascularization of the necrotic area in a clinical setting. We analyzed the concentrations of VEGF in the supernatant of MSCs. We observed an accumulation of minor amounts of VEGF under control conditions over 48 hours. This indicates that MSCs from SCD patients are capable of producing vasculogenic cytokines and growth factors that might mediate endogenous bone regeneration.

## Discussion

The present study was the first 5-year clinical trial in Brazil addressing cell therapy of avascular osteonecrosis of the femoral head in sickle cell-ill patients. The current study demonstrated that pre-collapse stages of ONFH in SCD patients could be treated safely and efficiently with BMMCs enriched with stem/progenitor cells combined with minimally invasive decompression. The implantation of BMMCs significantly decreased hip pain and other joint symptoms, and prevented the progression of the disease towards later osteonecrotic stages or collapse of the femoral head in SCD patients.

In this study, we conducted a 60-month follow-up on the patients, since disease progression and collapse of the femoral head are usually found in this period [38]. The major study limitation is the fact that no control group with decompression alone or with conservative treatment was included. The current data were compared with the natural evolution of hip osteonecrosis in adults with SCD [30]. In fact, even the 3.4 % (3 out of 89) of the treated patients that did not achieve the expected satisfactory clinical result were markedly better than those left to the natural evolution of ONFH. Extensive reports have described that, without a specific intervention, 70 % to 90 % of clinically diagnosed ONFH cases in SCD patients will progress to collapse of the femoral head and to secondary osteoarthritis within a 5-year period, and they will consequently undergo arthroplasty [6, 7]. In a series of 75 symptomatic SCD hips with ONFH, Hernigou et al. [38] described that disease progression occurred regardless of the stage at the initial diagnosis or in the presence/absence of significant risk factors that could cause collapse, suggesting that conservative treatment procedures should be instituted early to prevent a poor outcome in this disease. In our study, sickle-ill patients with early-stage ONFH (Ficat stage 0 to IIB) were enrolled for treatment when necrosis does not yet involve the complete femoral head. Patients with Ficat stage 0/I showed significant improvement in pain and function, and none of them progressed clinically. Among patients with stage IIA/IIB, 35 hips (out of 38) were treated successfully, while the rest reported no change or progressive pain. Importantly, we found that all patients who were treated with decompression and implantation of BMMCs enriched with stem/progenitor cells did not show postoperative collapse of the femoral head during the 60-month follow-up. Our results indicate a reduction in pain severity and other joint symptoms associated with early stages of ONFH with this treatment, and, at least for the 60-month follow-up period, disease progression remained stable.

The prevalence of osteonecrosis in patients with SCD is high, reaching up to 50 % of these individuals by 35 years of age, and their occupational and physical activity is substantially limited [6, 38, 39]. Several strategies have been examined to preserve rather than replace the femoral head and articular cartilage in SCD patients with osteonecrosis.

In our study, implantation of BMMC concentrate with minimally invasive decompression was used for all patients. Decompression with small-diameter 3-mm trephine had to be used to avoid leakage of the implanted BMMC population. This technique was developed to be less invasive and to reduce the risk of cartilage damage [40].

Avascular ONFH in SCD patients is a condition that progresses slowly, and for which no effective therapeutic option currently exists. Early in the course of the disease, core decompression remains the most logical treatment modality if one accepts that the condition is a compartment syndrome [40]. In an updated systematic review, Martí-Carvajal et al. [41] identified only one randomized prospective clinical study for osteonecrosis related to SCD. The report failed to show that core decompression alone or combined with physical therapy halted disease progression, improved clinical symptoms or improved quality of life in people with SCD [42]. Thus, despite several case series describing the utility of this procedure and its widespread use in clinical practice, the possible beneficial effects of hip core decompression alone for ONFH related to SCD remain inconsistent.

Since small-diameter decompression therapy alone does not stop the frequent progression to subchondral fracture of the femoral head in sickle-ill patients [42–44], our data suggest that implantation of BMMC concentrate with a minimally invasive technique might be effective in preventing the natural evolution to collapse of ONFH and will help avoid joint replacement in SCD patients. A recent study by Al Omran [44] compared the efficacy of decompressive methods for the treatment of pre-collapse ONFH in a consecutive series of SCD patients. He observed significant improvement in pain at the end of 2 years for Ficat stage I patients, but the clinical failure rate, as measured as progression to end-stage ONFH requiring hip arthroplasty, still reached 20–50 % in stage IIA/IIB patients. Thus, core decompression alone can delay the natural progression to collapse in SCD patients but still has a high failure rate without bone marrow grafting.

A small number of clinical studies of autologous BMMC implantation for early ONFH in SCD patients has been carried out. The original prospective study [19] of 189 hips treated with core decompression and autologous bone marrow concentrate has proved very beneficial in identifying the procedure and the number of injected progenitor cells needed for a better outcome. However, the influence of etiologic factors on the outcome was not demonstrated since the majority of ONFH in these patients was idiopathic, alcohol induced, or steroid related. Our findings are consistent with those reported by Hernigou et al. [30] who found greater clinical improvement, decreased pain, delayed progression of the disease and no subchondral fracture of the femoral head in sickle-ill patients treated with minimally invasive decompression and autologous bone marrow concentrate. The efficacy of BMMC implantation into ONFH was reported for all the patients postoperatively during a period as long as 17 years with the longest follow-up and with an average period of 10 years for 87 % of the hips [30].

Other studies that included pre-collapse ONFH patients, with diverse underlying pre-operative etiologies, have reported satisfactory decreases in pain and joint symptoms in patients treated with core decompression and implantation of BMMCs [15, 45, 46]. However, these studies did not prevent the evolution to the fracture stage between 24 and 60 months of follow-up. Indeed, in the bone marrow-grafted hip, one or more hips progressed to stage 3 during the follow-up period. In 2011, Gangji et al. [17] in a prospective randomized controlled trial reported that 8 of 11 hips in the core decompression group alone progressed to fracture and collapse, while in the core decompression and BMMC group only 3 of 13 progressed to collapse at the 5-year follow-up period. In a recent prospective trial, Ma et al. [47] looked at a similar group of patients (53 hips) with Ficat stage I to III ONFH. After a 2-year follow-up period there was a significant relief in pain (*P* < 0.05) and clinical joint symptoms in the core decompression and BMMC treatment group compared to the core decompression group alone. In addition, a significant number of hips in the core decompression group have deteriorated to the next stage after 24 months post-procedure compared to the BMMC treatment group.

Our study also showed promising biological properties of BMMCs enriched in stem/progenitor cells to be used for the treatment of early stages of OHFH related to SCD. We demonstrate that ex vivo expanded MSCs from SCD patients maintained the cell replicative capacity without significant loss of their specific biomolecular characteristics, differentiation capacity into multiple mesenchymal phenotypes, and osteogenic differentiation activities. Furthermore, the presence of vasculogenic cytokines and growth factors (IL-8, TGF-β, SDF-1α and VEGF) in conditioned medium from MSCs reinforce the beneficial effects for cell therapy of ONFH in SCD patients.

The rational for a cell-based strategy for the treatment of ONFH is to provide stem cells and other progenitor cells to potentially improve the local environment in the affected hip. In SCD, however, previous studies have not focused on the detailed content of stem/progenitor cell populations in the infused BMMCs or have not addressed the potential of the cells isolated from SCD patients [19, 30]. In our study, we further characterized the heterogeneous group of the bone marrow stem cell population isolated from SCD patients with ONFH. FACS analyses of the infused BMMCs have shown enriched cell populations with surface markers characteristic of endothelial progenitors and hematopoietic stem cells, as compared to mononuclear cells in the native BMA of SCD patients. Furthermore, the number of CFU-F and CFU-O produced in BMMCs, which are representative of the total number of connective progenitors, were significantly higher as compared to native BMA. Thus, it can be assumed that the infused BMMCs contained an enriched mixture of MSCs and endothelial and hematopoietic progenitor cells.

The efficacy of the cell-based therapeutic approaches to enhance tissue repair in ONFH is based on the frequency and activity of MSC content in the grafted BMMCs [12]. However, there is evidence that the replicative capacity and osteogenic activity of MSCs in the bone marrow are decreased in non-traumatic ONFH [19–21, 23]. Hernigou and Beaujean described that the number of both granulocyte-macrophage progenitor cells in the hematopoietic compartment and fibroblast colony-forming units in the stromal compartment of the bone marrow of the iliac crest were decreased in patients who were receiving corticosteroids or who abused alcohol [22]. In addition, a more recent comparative analysis of the osteogenic and growth potential of MSCs in patients with ONFH has shown differences according to the risk factors [21]. In our study, the obtained data demonstrate that the replicative activities, osteogenic differentiation abilities and production of cytokines and growth factors in MSCs isolated from the iliac crest are not impaired in sickle cell-ill patients with early stages of ONFH. Moreover, the ex vivo expanded MSCs maintained cell replicative capacity without significant loss of their specific biomolecular characteristics and differentiation potential into multiple mesenchymal phenotypes. These findings extend the notions that MSCs from SCD patients, either in BMMCs or ex vivo expanded in culture, represent an ideal cell type to mimic a bone autograft, containing all the key components required for bone repair in pre-collapse stages of ONFH.

We are aware that our experimental design has several limitations. The presented data is not a controlled study, and no parallel control groups of decompression alone and conservative treatments were included. SCD is a systemic life-threatening hemoglobinopathy associated with a high prevalence of comorbidities, and SCD patients are at more risk of intra- and post-operative complications and suboptimal outcome of complex orthopedic procedures [48–50]. The high failure rate of core decompression alone and the potential risk of harm during surgical intervention for SCD patients allocated to a control group predict a poor prognosis for these patients. This stands in contrast to the safety and efficiency confirmed by a long-term follow-up of patients receiving the cell therapy. The inclusion of a control group was not in accordance with the ethical recommendations of the national authorities that have analyzed and provided the financial support for this study. Quite clearly, prospective randomized studies including a comparison with an active control group would be nevertheless desirable in order to determine more comprehensively the efficacy of the treatment described in this study.

Another limitation is consequent to the fact that only a few patients underwent MRI during the follow-up, and we were unable to monitor bone formation in the necrotic area or examine how this may have affected outcomes. Future studies should incorporate such monitoring. We also cannot be certain whether the grafted BMMCs remained at the site of implantation and/or participated in mobilization of resident or circulating mesenchymal cells into the lesion. The BMMC solution was completely absorbed by the bone-filling material and the cells may have found an autologous environment similar to the bone marrow from which they have been obtained.

We used MSCs expanded from SCD patients with the very early stages of ONFH (Ficat 0) as a comparison group to MSC expanded from later stages (Ficat I, II) because of the ethical difficulties in using bone marrow samples from SCD patients without an overt involvement of the skeletal tissues. Most clinical studies investigating the biological properties of MSCs have demonstrated that the number of CFU-F in the iliac crest of control patients without osteonecrosis is equivalent to the patients with pre-collapse stages in ONFH secondary to SCD [11, 30]. Moreover, there is evidence that active new bone formation occurs in femoral heads around necrotic areas in most SCD patients with ONFH when compared to a “low-risk” osteonecrosis cohort of patients [50, 51].

We are aware that the differentiation of MSCs is highly dependent upon in vivo and in vitro conditions, and our in vitro findings may not reflect the potential of MCS in an in vivo environment. Yoo and coworkers demonstrated that ex vivo expanded MSCs isolated from the iliac crest of patients from idiopathic or alcohol-induced ONFH have similar proliferative activities and osteogenic differentiation abilities compared with those of patients with a non-necrotic hip disorder [52]. Therefore, for cell-based strategies, freshly prepared BMMCs enriched with MSCs may better represent the in vivo condition and the data demonstrated in BMMCs may better be related to satisfactory clinical outcomes. However, we also consider that interpersonal variations and potential experimental bias are likely to increase when assays are conducted separately using freshly prepared cells.

Our study did not allow study of the relationship between the clinical outcome and the number or phenotypic profile of cells implanted per volume of lesion. We found substantial donor-dependent variations and high variability in terms of CFU-F and CFU-O within the SCD BMMCs. Similarly, Siddappa and coworkers have noted osteogenic variability in human BMMCs as a result of sampling method and cellular heterogeneity among the donor population [53]. So far, we have been unable to define which cells might be responsible for the therapeutic effects in the mixture of stem cell populations in the grafted BMMCs. Moreover, some of the cells might have leaked through the trephine, although the greatest part of the bone marrow remained in the area of osteonecrosis. Another possible explanation for the therapeutic effect is that injected BMMCs secreted and supplied osteogenic and angiogenic factors resulting in increased osteogenesis (i.e., TGF-β, IL-8) [13, 54] and angiogenesis (i.e., VEGF, SDF-1α) that would create sufficient bone repair capacity. Further studies must be carried out to confirm the clinical effectiveness of this technique with a longer follow-up time in comparison to standard surgical treatment.

## Conclusion

Our clinical and laboratory findings indicate that the replicative abilities, osteogenic potentials and secretion of osteogenic/angiogenic factors by MCSs are not impaired in SCD patients with early stages of ONFH. They also indicate that minimally invasive implantation of autologous BMMCs enriched with progenitor/stem cells is a promising strategy for the treatment of ONFH and orthopedic pathologies related to SCD.

## Additional files

Additional file 1: Table S1. Radiological findings in modified Ficat and Arlet and Steinberg grading systems. Additional file 2: Table S2. Primer sequences used for quantitative reverse transcriptase polymerase chain reaction gene expression analysis. Additional file 3 : Figure S1. Frequency of CFU-F/10^6^ cells isolated in BMA and BMMC from individuals with osteonecrosis at a distant site. An average of 28.2 ± 13.9 CFU-F colonies were observed in BMMC (1.0 × 10^6^ cells/25 cm^2^) cultures compared to 8.4 ± 5.3 colonies (1.0 × 10^6^ cells/25 cm^2^) in the BMA group The average concentration of CFU-F in BMMCs compared to the initial BMA (ratio BMMC/BMA) was 3.3-fold. ****P* < 0.0001. Additional file 4: Figure S2. Flow cytometry analysis of MSC cultures isolated from SCD patients with pre-collapsed ONFH. MSCs were isolated and expanded from age-matched BMMC samples. No significant differences in surface marker expression were found in MSCs between hemoglobin genotype groups (a), number of passages in culture (b) or disease stage groups (c). Data represent mean ± SD of three different experiments.

## Abbreviations

ALPL: alkaline phosphatase
BMA: bone marrow aspirate
BMMC: bone marrow-derived mononuclear cells
CFU-F: colony-forming unit fibroblast
CFU-O: colony-forming unit osteoblast
COL1A1: collagen, type I, alpha 1
DMEM: Dulbecco’s modified Eagle’s medium
ENG: endoglin
EPC: endothelial progenitor cell
FITC: fluorescein isothiocyanate
GAPDH: glyceraldehyde-3-phosphate dehydrogenase
HbS: hemoglobin S
HHS: Harris hip score
HPRT1: hypoxanthine phosphoribosyltransferase 1
IL: interleukin
MRI: magnetic resonance imaging
MSC: mesenchymal stromal cell
ONFH: osteonecrosis of femoral head
PE: phycoerythrin
PerCP: peridinin chlorophyll protein complex
RN18S1: RNA, 18S ribosomal 1
RUNX2: Runt-related transcription factor 2
SCD: sickle cell disease
SDF: stromal cell-derived factor
TGF: transforming growth factor
VEGF: vascular endothelial growth factor
