# Supplementary material for: Efficacy of autologous stem cell-based therapy for osteonecrosis of the femoral head in sickle cell disease: a five-year follow-up study
Source: Stem Cell Res Ther. 2015 May 29;6(1):110. doi: 10.1186/s13287-015-0105-2 (PMC4465459; doi:10.1186/s13287-015-0105-2)
Supplement: Additional file 1: Table S1. — Radiological findings in modified Ficat and Arlet and Steinberg grading systems. [file 13287_2015_105_MOESM1_ESM.pdf]

Additional File 1

Table 3 - Radiological findings in modified Ficat & Arlet and Steinberg grading system

Stage	Radiograph	Pain	MRI
Stage 0 (Preclinical)	Normal	Nil to weak	Normal
Stage I (Preradiographical)	Normal or minor osteopaenia	Mild pain in the affected hip<br>Pain with internal rotation	Oedema
Stage IIA	Femoral head sclerosis, porosis or cysts, normal contour and joint line	Mild and worsening pain<br>Stifness	Diffuse<br>Focal lesions
Stage IIB	"Crescent sign" (subchondral fracture)<br>Flattened areas of femoral head	Worsening persistent pain<br>Radiation to knee and limp	Same as X-Ray
Stage III	Loss of sphericity of the head<br>Collapse, joint space normal	Moderate to severe<br>Radiation to knee and limp	Same as X-Ray
Stage IV	Reduction of articular space, acetabular changes	Moderate to severe<br>Limp	Same as X-Ray

Abbreviation: MRI, magnetic resonance imaging.
