# Supplementary material for: Efficacy of autologous stem cell-based therapy for osteonecrosis of the femoral head in sickle cell disease: a five-year follow-up study
Source: Stem Cell Res Ther. 2015 May 29;6(1):110. doi: 10.1186/s13287-015-0105-2 (PMC4465459; doi:10.1186/s13287-015-0105-2)
Supplement: Additional file 2: Table S2. — Primer sequences used for quantitative reverse transcriptase polymerase chain reaction gene expression analysis. [file 13287_2015_105_MOESM2_ESM.pdf]

Additional File 2

Table 4 - Primer sequences used for quantitative reverse transcriptase–polymerase chain reaction gene expression analysis

Gene	5'to 3'	Primers
ALPL	Forward	AGCCCAGAGATGCAATCG
	Reverse	CTATCCTGGCTCCGTGCTC
COL1A1	Forward	CACACGTCTCGGTCATGGTA
	Reverse	AAGAGGAAGGCCAAGTCGAG
ENG	Forward	CTGAGGACCAGAAGCACCTC
	Reverse	TCCATGTCCTCTTCCTGGAG
RUNX2	Forward	CCATAACCGTCTTCACAAATCC
	Reverse	AATGCGCCCTAAATCACTG
GAPDH	Forward	ACCCACTCCTCCACCTTTGA
	Reverse	CTGTTGCTGTAGCCAAATTCGT
HPRT1	Forward	GAAGTCTTGCTCGAGATGTGA
	Reverse	TCCAGCAGGTCAGCAAAGAAT
RN18S1	Forward	GACTTCACGCAAGCCTATGAC
	Reverse	CTGTGATGCCCTTAGATGTCTG

Abbreviations: ALPL: alkaline phosphatase; COL1A1 collagen, type I, alpha 1; ENG: Endoglin; RUNX2: Runt-related transcription factor 2; GAPDH Glyceraldehyde 3-phosphate dehydrogenase; HPRT1: hypoxanthine phosphoribosyltransferase 1; RN18S1: RNA, 18S ribosomal 5
