# Supplementary figures and images for: Efficacy of autologous stem cell-based therapy for osteonecrosis of the femoral head in sickle cell disease: a five-year follow-up study
Source: Stem Cell Res Ther. 2015 May 29;6(1):110. doi: 10.1186/s13287-015-0105-2 (PMC4465459; doi:10.1186/s13287-015-0105-2)

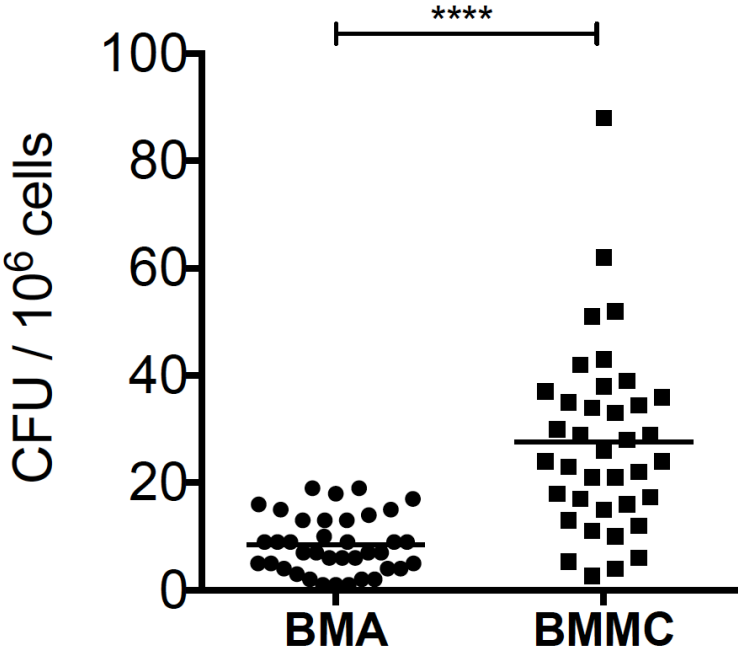

Supplement: Additional file 3 : Figure S1. — Frequency of CFU-F/106 cells isolated in BMA and BMMC from individuals with osteonecrosis at a distant site. An average of 28.2 ± 13.9 CFU-F colonies were observed in BMMC (1.0 × 10^6^ cells/25 cm2) cultures compared to 8.4 ± 5.3 colonies (1.0 × 10^6^ cells/25 cm2) in the BMA group The average concentration of CFU-F in BMMCs compared to the initial BMA (ratio BMMC/BMA) was 3.3-fold. ***P < 0.0001. [file 13287_2015_105_MOESM3_ESM.pdf]

A

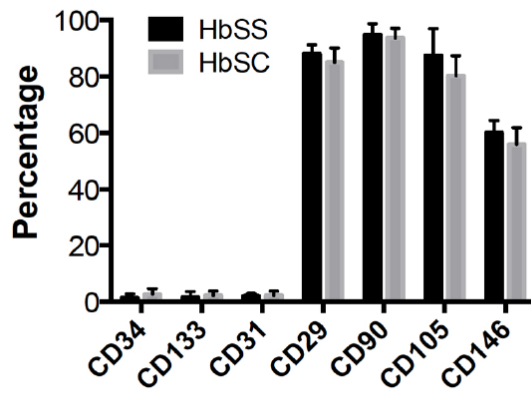

B

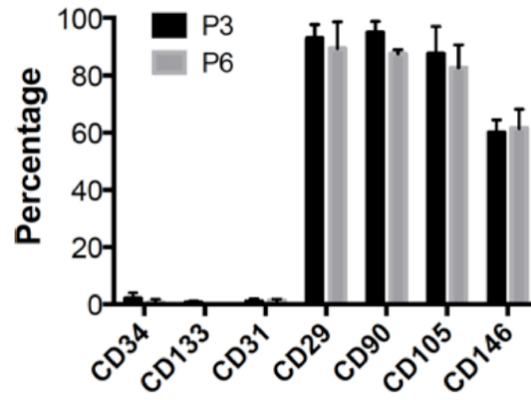

C

|       | FICAT 0   | FICAT II  |
|-------|-----------|-----------|
| CD34  | 4,0 ±0,6  | 0,7 ±0,8  |
| CD133 | 6,9 ±1,5  | 0,4 ±0,5  |
| CD31  | 0,6 ±1,0  | 1,8 ±1,2  |
| CD29  | 93,5 ±4,5 | 87,2 ±7,1 |
| CD90  | 96,9 ±3,5 | 90,6 ±4,2 |
| CD105 | 96,2 ±8,6 | 81,4 ±6,9 |
| CD146 | 60,0 ±4,0 | 58,6 ±6,4 |

Supplement: Additional file 4: Figure S2. — Flow cytometry analysis of MSC cultures isolated from SCD patients with pre-collapsed ONFH. MSCs were isolated and expanded from age-matched BMMC samples. No significant differences in surface marker expression were found in MSCs between hemoglobin genotype groups (a), number of passages in culture (b) or disease stage groups (c). Data represent mean ± SD of three different experiments. [file 13287_2015_105_MOESM4_ESM.pdf]
